# Multi-Sensor Mobile Laser Doppler Vibrometry for Internal Damage Detection in Reinforced Concrete Structures

**DOI:** 10.3390/s26175612

**Published:** 2026-09-03

**Authors:** Shichuan Liang, Dejin Zhang

**Affiliations:** 1 School of Civil and Transportation Engineering, Shenzhen University, Shenzhen 518060, China; 2250471003@email.szu.edu.cn; 2 Guangdong Key Laboratory of Urban Informatics, Shenzhen University, Shenzhen 518060, China; 3 School of Architecture and Urban Planning, Shenzhen University, Shenzhen 518060, China

**Keywords:** infrastructure void detection, vibration analysis, laser doppler vibrometer (LDV), mobile measurement, neural network

## Abstract

Reinforced concrete structures are critical components of modern infrastructure, and the detection of internal damage within these structures has long been an important research topic. These damages can be detected using the vibration response of the structures, and laser Doppler vibrometry (LDV) on a moving platform offers an efficient, long-range sensing approach for structural vibration monitoring. However, extending LDV-based damage detection from static to mobile measurement requires addressing the effects of measurement signal frequency shift, platform vibrations, and speckle noise. To address these issues, this paper proposes a mobile measurement damage detection framework that integrates multi-source information with artificial intelligence algorithms. First, theoretical derivation and numerical simulation demonstrate that the vibration frequency shift induced by moving speed is negligible, proving that mobile and static measurement signals are similar in both time and frequency domains. Then, a multi-sensor data processing framework is used to decouple the platform vibration and suppress speckle noise. Finally, a spatial-aware CNN network is employed to achieve damage detection under mobile measurement. The results reveal that the vibration signals for large-scale voids were effectively recovered, whereas signals for small-scale voids and healthy regions were only partially recovered. Voids with a tested size of 0.4 m and larger were successfully identified under the experimental conditions. The results demonstrate the feasibility of extending static LDV-based void detection to mobile measurement, providing a theoretical and technical basis for efficient, non-contact mobile inspection of infrastructure.

## 1. Introduction

Reinforced concrete structures are critical components of modern infrastructure, such as highways, tunnel linings, ballastless tracks, and bridge piers. They play a key role in socioeconomic development and transportation. Due to different construction methods, construction sequence, and material properties, natural bonding interfaces inevitably exist within these structures. During long-term service, cyclic loading, environmental erosion, and material aging can cause cracks and voids to develop at these interfaces [1,2]. Typical examples include cement–asphalt (CA) mortar void in ballastless tracks [3], void between primary support and secondary lining in tunnel vaults [4], and interface debonding in concrete-filled steel tubes [5]. These defects can weaken the strength of structures and accelerate structural damage caused by external environmental factors, posing a severe threat to structural safety. Therefore, timely detection and repair of internal damage in reinforced concrete structures are crucial for ensuring safe operation and reducing maintenance costs for large-scale infrastructure.

Vibration response testing is a traditional technique for damage identification [6,7]. When internal defects occur, the local stiffness of the structure changes. This alters the vibration characteristics generated by local impact, allowing effective detection of voids through response analysis. It has been applied to concrete void detection, bolt loosening detection, and other structural inspection tasks [8,9]. In the early stages of impact vibration testing, void detection relied heavily on experienced personnel by listening to impact sounds. This method was highly subjective and required high operator expertise. With advances in sensor technology, piezoelectric accelerometers allowed researchers to measure surface vibration directly and conduct quantitative spectral analysis. However, this contact-based measurement technique resulted in very low inspection efficiency. Some researchers have found that the sound radiated into the air during concrete vibration has similar characteristics to the vibration itself. Based on this finding, they proposed an air-coupled approach that uses microphones to capture impact sounds. This method enables non-contact measurement and improves inspection efficiency [10]. It has been widely applied to detect voids in pavements and concrete structures, often paired with chain-drag or rotary-impact devices to achieve mobile inspection [11,12,13].

However, many studies on concrete void detection show that impact sound signals fail to detect deep internal voids [14,15]. Deep voids provide higher flexural rigidity, which results in smaller out-of-plane deflection by impact. This small movement cannot sufficiently compress the air to generate effective acoustic radiation [16]. As a result, the acoustic signals from structural vibration are masked by the initial impact noise, making deep voids undetectable via impact sound signals. Several studies reveal that impact vibration signals at deep voids still follow distinct patterns as void severity changes [17,18]. Although ref. [18] demonstrated the feasibility of LDV-based non-contact vibration measurements for ballastless track void detection under static conditions, practical application of this technique to large-scale infrastructure inspection remains limited by the low measurement efficiency associated with point-by-point stationary measurements. To address this limitation, this study extends the static measurement to a mobile platform and investigates the questions of mobile measurement.

LDV is a non-contact sensor based on the Doppler effect for measuring vibration velocity. It can detect tiny surface vibrations from a long distance, providing a new approach for long-range, non-contact vibration monitoring of infrastructure. Some researchers have integrated LDV into vehicles or unmanned aerial vehicles (UAVs), for non-contact inspection of elevated or distant structures, such as road deflection and bridge piers [19,20]. However, the raw signals collected during mobile measurements inevitably contain platform vibration and speckle noise. These disturbances can mask the actual structural vibration signals. Additional devices, such as accelerometers and inertial measurement units (IMUs), are often required to separate platform-induced disturbances from the measured signals. Specific methods are also needed to suppress speckle noise. In addition, the impact vibration response of a structure is composed of multiple vibration modes. Analyzing the frequency spectrum of the vibration signal helps identify excited modes and determine the presence of defects. During static measurement, the measuring point remains fixed, and the recorded signal represents the temporal evolution of mode shapes at that point. In contrast, under mobile measurement, the sensor point continuously moves forward along the platform path. The resulting signal becomes a time–space-coupled continuous vibration response along the spatial trajectory. Whether vibration signals collected during movement can reflect damage modes as accurately as static signals, and whether they share the same equivalence and sensitivity to damage severity, still lacks systematic mechanism exploration and experimental validation.

In recent years, artificial intelligence methods have been widely applied to vibration-based structural health monitoring. Unlike conventional approaches that rely on manually extracted indicators, data-driven methods can learn complex nonlinear relationships between vibration responses and structural damage directly from measured data [21,22]. Meanwhile, these methods have also been used for signal reconstruction and denoising to recover structural response signals from measurements contaminated by environmental noise, sensor noise, or other disturbances [23,24,25]. These capabilities are perfectly suited to the problems that this article aims to solve.

Based on the above analysis, this paper proposes a non-contact detection framework for internal damage in concrete structures based on a mobile Doppler platform. The framework uses a laser Doppler vibrometer mounted on a mobile platform to measure structural surface vibrations in a non-contact manner during movement. A data processing framework consisting of platform compensation and a UNet-based signal reconstruction method is proposed to suppress noise. The similarities and differences between the vibration characteristics obtained from mobile and static measurements are further investigated. Finally, a spatial-aware CNN is used to achieve void detection. The results show that the proposed method can accurately identify large-scale voids within concrete structures during mobile measurements. This method provides a new approach for intelligent infrastructure maintenance and structural health monitoring.

## 2. Materials and Methods

### 2.1. Detection Principle

In actual concrete infrastructure, due to material degradation and the shape of void regions during the service life, there are always deviations between actual vibration parameters and theoretical values. Moreover, the primary objective of our paper is to identify voids using the patterns of vibration responses, rather than specific modal parameter values. Therefore, in this paper, a simpler model of a simply supported plate is used to describe the vibration patterns, making them easier to understand. According to the plate vibration theory, the deflection response of the impact response at a certain point at the structural defect under static measurement can be simplified and written as [26,27]:

(1) $$\begin{array}{cc} w(x,y,t) & =\sum_{m}\sum_{n}\frac{IW_{mn}\left(x_{0},y_{0}\right)}{{\bar{\omega }}_{mn}M_{mn}}W_{mn}\left(x,y\right)e^{-\xi_{mn}\omega_{mn}t}\sin{\bar{\omega }}_{mn}t \end{array}$$

(2) $$\begin{array}{cc} W_{mn}\left(x,y\right) & =\sin\frac{m\pi x}{a}\sin\frac{n\pi y}{b} \end{array}$$

(3) $$\begin{array}{cc} \omega_{mn} & =\pi^{2}\left(\frac{m^{2}}{a^{2}}+\frac{n^{2}}{b^{2}}\right)\sqrt{\frac{D}{\rho h}} \end{array}$$

(4) $$\begin{array}{cc} {\bar{\omega }}_{mn} & =\omega_{mn}\sqrt{1-\xi_{n}^{2}} \end{array}$$

where w(x,y,t) is the deflection at the measured position (x,y) on the structure surface, m,n are the modal orders in the x,y directions, $W_{mn}$, $\omega_{mn}$, $M_{mn}$, $\xi_{mn}$ are the (m,n)th order mode shape, frequency, mass and damping respectively. $\left(x_{0},y_{0}\right)$ is the impact position, *I* is the impulse generated by the impact. a,b are the length and width of the damaged plate, *D* is the flexural stiffness of the plate, ρ is the density of the plate, and *h* is the thickness of the plate. When the degree of damage increases, the dimensions a,b of the structure above the void become larger, resulting in the natural frequency $\omega_{mn}$ becoming smaller. Based on this change, the void can be effectively identified.

During the movement, the measurement position moves with the platform. Assuming that the platform moves forward along the *x* axis at a speed *v*, the actual measured signal is:

(5) $$\begin{array}{cc} w(x+vt,y,t) & =\sum_{m}\sum_{n}\frac{IW_{mn}\left(x_{0},y_{0}\right)}{{\bar{\omega }}_{mn}M_{mn}}W_{mn}\left(x+vt,y\right)e^{-\xi_{mn}\omega_{mn}t}\sin{\bar{\omega }}_{mn}t\\ \; & =\sum_{m}\sum_{n}\frac{IW_{mn}\left(x_{0},y_{0}\right)}{{\bar{\omega }}_{mn}M_{mn}}\sin\frac{n\pi (x+vt)}{a}\sin\frac{m\pi y}{b}e^{-\xi_{mn}\omega_{mn}t}\sin{\bar{\omega }}_{mn}t \end{array}$$

According to this formula, the impact response of the structure will change with the coordinates in the *x* and *y* directions. However, when the measurement point moves along the *x* axis, the *y* axis mode shape remains unchanged, and the measured vibration signal will only be affected by the mode shape in the *x* direction. Therefore, this equation can be simplified. A one-dimensional beam is considered as an example to analyze the variation in the measured signal during mobile measurements. Consider a simply supported beam at both ends. The beam has a length of *L*, a flexural stiffness of EI, and a mass per unit length of ρA. Its impact-induced vibration response can be expressed in a similar form as follows:

(6) $$\begin{array}{cc} w(x,t) & =\sum_{n}\frac{I\phi_{n}\left(x_{0}\right)}{M_{n}{\bar{\omega }}_{n}}e^{-\xi_{n}\omega_{n}t}\phi_{n}\left(x+vt\right)\sin{\bar{\omega }}_{n}t \end{array}$$

(7) $$\begin{array}{cc} \phi_{n}\left(x\right) & =\sin\frac{n\pi x}{L} \end{array}$$

(8) $$\begin{array}{cc} \omega_{n} & ={\left(\pi \frac{n\pi }{L}\right)}^{2}\sqrt{\frac{EI}{\rho A}} \end{array}$$

(9) $$\begin{array}{cc} {\bar{\omega }}_{n} & =\omega_{n}\sqrt{1-\xi_{n}^{2}} \end{array}$$

For static measurements, the measurement velocity is v=0. The measured signal therefore vibrates in the form of $\sin{\bar{\omega }}_{n}t$, and the frequency observed in the signal spectrum corresponds to the natural frequency of the structure. In mobile measurements, however, the vibration response contains both the velocity-dependent term $\phi_{n}\left(x+vt\right)$ and the frequency term $\sin{\bar{\omega }}_{n}t$. Using the product-to-sum identity for trigonometric functions, the signal can be modulated as follows:

(10) $$\begin{array}{c} \phi_{n}\left(x+vt\right)\sin{\bar{\omega }}_{n}t=\frac{1}{2}\cos\left(\left({\bar{\omega }}_{n}-\frac{n\pi v}{L}\right)t-\frac{n\pi x}{L}\right)-\frac{1}{2}\cos\left(\left({\bar{\omega }}_{n}+\frac{n\pi v}{L}\right)t+\frac{n\pi x}{L}\right) \end{array}$$

Therefore, under mobile measurement conditions, for each excited mode, its natural frequency ${\bar{\omega }}_{n}$ will be split into two symmetrical side frequency peaks ${\bar{\omega }}_{n,1}$ and ${\bar{\omega }}_{n,2}$ on the spectrum, which are related to the moving speed and modal order. The impact of these two factors on the measurement signal needs to be discussed.

(11) $$\begin{array}{c} {\bar{\omega }}_{n,1}={\bar{\omega }}_{n}-\frac{n\pi v}{L},{\bar{\omega }}_{n,2}={\bar{\omega }}_{n}+\frac{n\pi v}{L} \end{array}$$

### 2.2. Experimental Equipment

**Figure 1:**
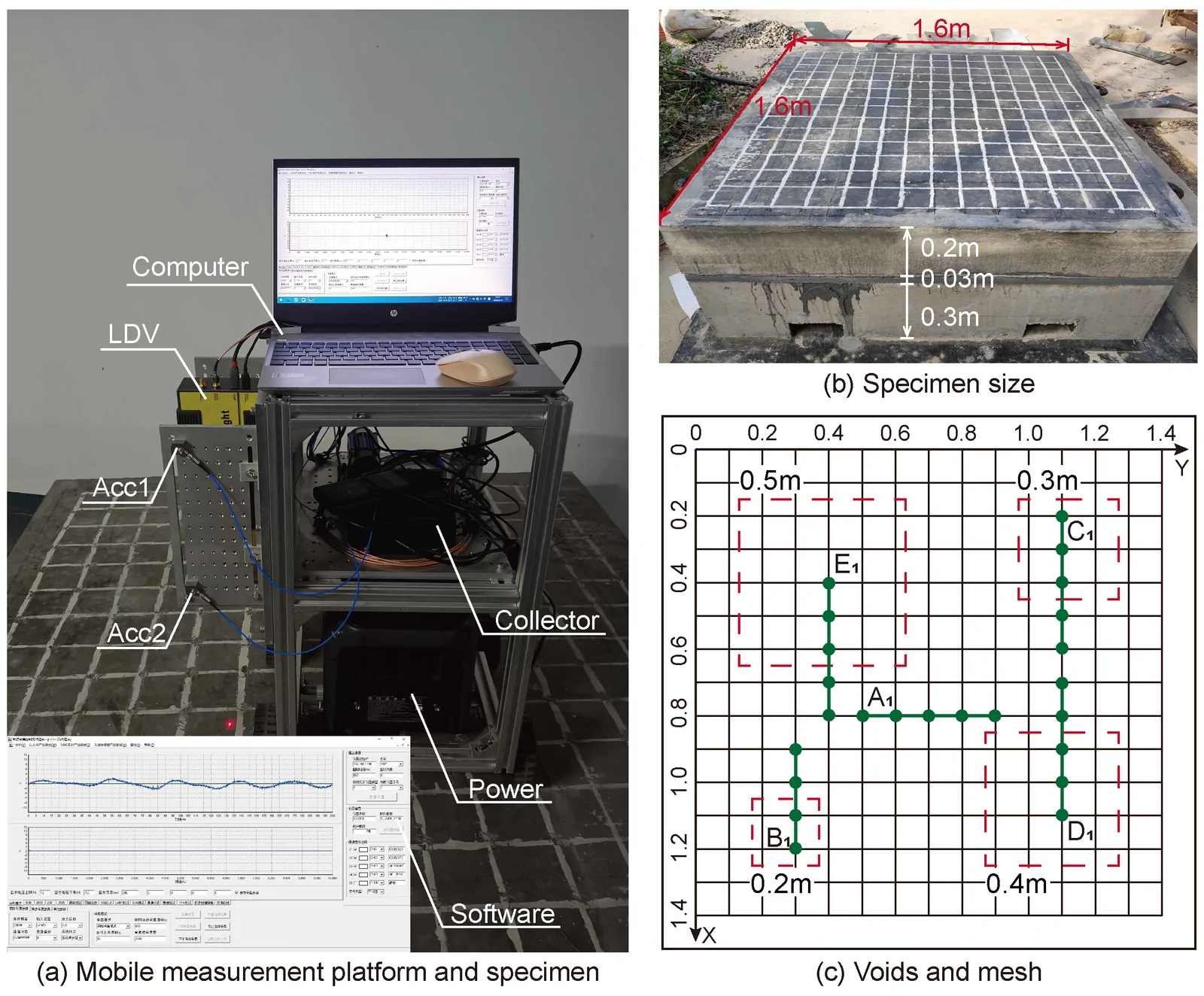
Experimental environment (the green dots are the measurement points used in Figure 3; the red dashed lines indicate the actual void position).

To investigate the variation characteristics of mobile measurement signals and the problems encountered in using a mobile Doppler platform to detect voids, comprehensive validation was conducted through theoretical analysis, numerical simulations, and experimental measurements. The concrete specimen used in this study is a scaled ballastless track structure [18]. The specimen retains full-scale vertical dimensions matching those of a real track structure, consisting of three layers: a track slab, a mortar layer, and a concrete base. Its transverse dimensions are reduced. Four voids of varying dimensions are embedded in the mortar layer, and a 10 cm grid is meshed on the structural surface. Detailed dimensions and locations are shown in Figure 1. The measurement system consisted of a mobile platform, an LDV, two accelerometers, a data collector, and a laptop computer. The data collector achieves synchronized acquisition of signals from both the vibrometer and the accelerometers. In the experiment, 30 impacts are performed at each grid point firstly using a static measurement method. During the mobile measurements, impacts were applied as the laser spot moved to the predefined grid points along the survey line in *x* axis, as closely as possible, so that the actual measurement locations could also be maintained near the grid points. This strategy was adopted to mitigate the positioning issue, which cannot be fully resolved in the current study. Each survey line is measured 20 times.

### 2.3. Framework

**Figure 2:**
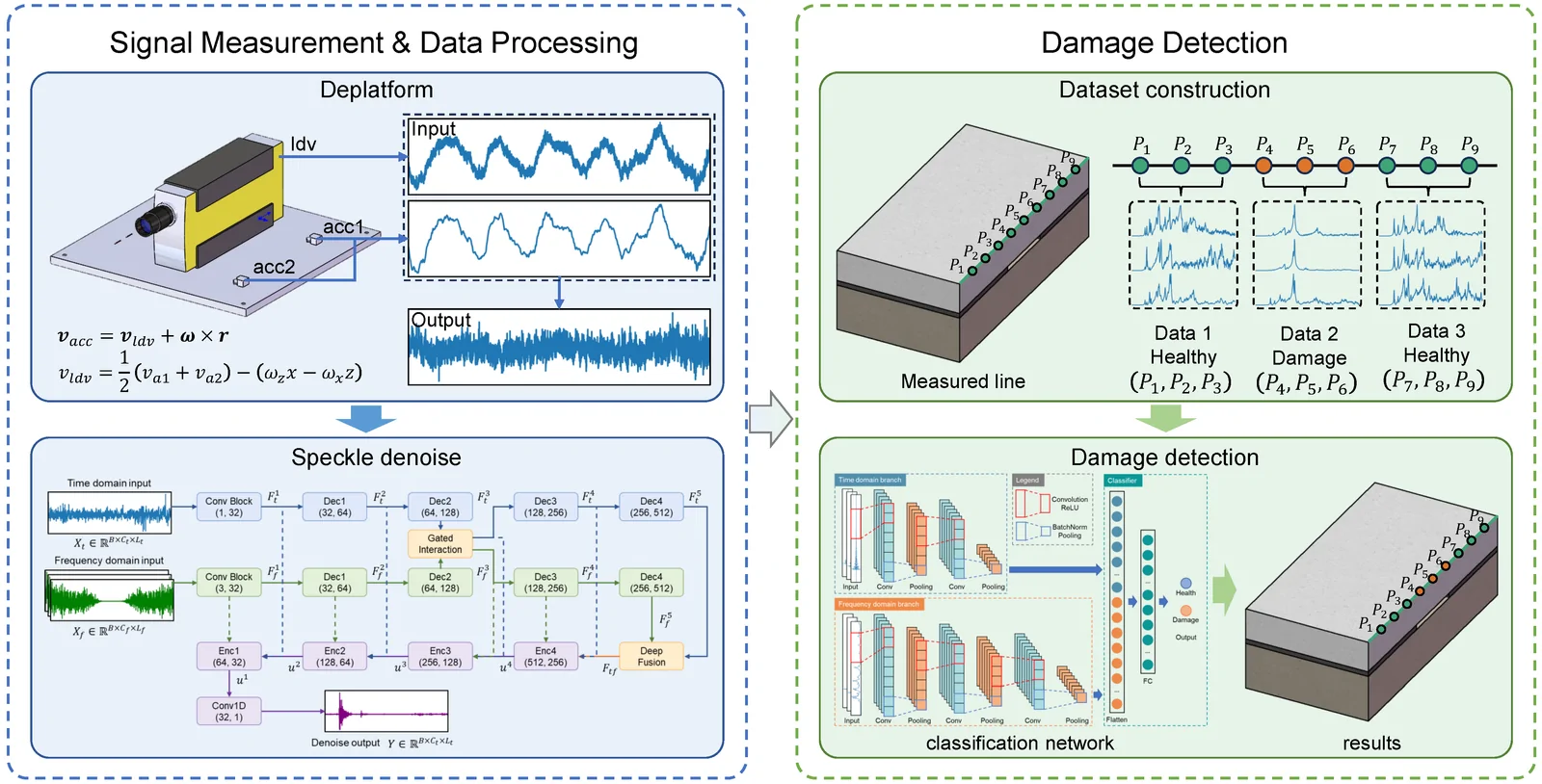
Damage detection framework.

To achieve efficient detection of internal damage in concrete structures using a mobile Doppler vibration measurement platform, this paper develops a comprehensive framework for mobile measurement and damage identification, as shown in Figure 2. The framework consists of two core components: signal measurement with data processing, and damage detection. The former uses multi-sensor cooperative measurement and signal processing to suppress mobile platform vibration and noise generated during measurement, thereby restoring the structural impact vibration response from raw measurement signals. The latter uses the processed vibration signals to extract damage-related features and achieve internal damage identification. This paper focuses primarily on the overall feasibility of the proposed mobile detection framework, systematically analyzes variations in structural impact vibration responses under mobile conditions, and the reconstruction effect of data processing on the mobile measurement signal. Furthermore, it validates the effectiveness of processed mobile measurement signals in damage identification. Therefore, for the steps such as signal acquisition, platform motion compensation, noise suppression, and damage identification models, this paper presents only their basic principles and implementation workflows to maintain the completeness of the research methodology and experimental results.

#### 2.3.1. Signal Measurement and Data Processing

Platform vibration and speckle noise are inevitable during mobile measurements using vibrometers. Some researchers have developed methods to address noise in mobile measurements of railway sleeper vibration [28,29]. However, impact responses and speckle noise both exhibit short-duration and energy-concentrated characteristics. These methods may therefore mistakenly identify impact responses as speckle noise and remove useful vibration information. To address this problem, we developed a denoising method specifically for impact response measurements.

Step 1: Platform vibration compensation. Platform vibration data is collected using two triaxial accelerometers placed parallel to the laser line. Based on rigid body kinematics and the relative positions between the LDV and the accelerometers, the platform vibration signal along the LDV laser direction during platform movement is calculated. This signal is then subtracted from the raw LDV signal to eliminate platform vibration.

(12) $$\begin{array}{cc} v_{\mathrm{acc}} & =v_{\mathrm{ldv}}+\omega \times r \end{array}$$

(13) $$\begin{array}{cc} v_{\mathrm{acc}}^{1}-v_{\mathrm{acc}}^{1} & =\omega \times (r_{1}-r_{2}) \end{array}$$

(14) $$\begin{array}{cc} v_{\mathrm{acc}}^{1}+v_{\mathrm{acc}}^{1} & =2v_{\mathrm{ldv}}+\omega \times \left(r_{1}+r_{2}\right) \end{array}$$

Taking the LDV vibration velocity $v_{\mathrm{ldv}}$ as the reference, the velocity vectors of the two accelerometers, $v_{\mathrm{acc}}^{1}$ and $v_{\mathrm{acc}}^{2}$, can be expressed using their position vectors relative to the LDV. We are only concerned with the platform vibration along the laser beam direction. According to the working principle of the LDV, rotation around the laser beam direction does not affect the measurement data. Therefore, the LDV measurements are mainly affected by translational motion along the laser beam and rotations around the axes perpendicular to the laser beam. When the two accelerometers are collinearly aligned along the laser beam direction, the rotational components ω about the other two axes can be calculated using Equation (13). Then, summing the two accelerometer signals and removing the rotational component yields the isolated vibration velocity of the vibrometer. Since the LDV measure velocity signals, while accelerometers measure acceleration signals, it is necessary to integrate the signal from the accelerometer into the velocity domain. To avoid integration drift, a 5 Hz high-pass filter is applied to the accelerometer data both before and after integration.

Step 2: Speckle noise suppression. After platform vibration compensation, the measured signal still contains speckle noise. A dual-branch U-Net is therefore used to suppress the speckle noise. The platform-compensated signal is first divided into segments with a duration of 0.1 s. Fast Fourier transform (FFT) is then applied to each segment, and the spectrum is decomposed into three-channel data consisting of [real part, imaginary part, magnitude]. The single-channel time-domain signal $\left(X_{t}\in R^{1\times L_{t}}\right)$ is fed into the time-domain branch, while the three-channel frequency-domain signal $\left(X_{f}\in R^{3\times L_{f}}\right)$ is fed into the frequency-domain branch. Unlike traditional denoising methods, the network directly learns the target vibration to achieve signal reconstruction. The network consists of four encoder and decoder layers. Because time-domain and frequency-domain features are different, feature fusion and information exchange are performed between the two branches at intermediate encoder layers. This prevents the features from becoming isolated during the independent downsampling processes. The time-domain and frequency-domain features are further fused at the final layer. By learning signal features under complex physical environments, the network restores the true structural vibration signal to the greatest extent possible. This provides reliable data support for subsequent damage diagnosis. The loss function is

(15) $$\begin{array}{c} L=\frac{1}{N}\sum_{i=1}^{N}w_{i}{\left({\hat{s}}_{i}-s_{i}\right)}^{2}+\frac{1}{K}\sum_{k=1}^{K}\left|log\left({\hat{P}}_{k}\right)-log\left(P_{k}\right)\right| \end{array}$$

where *N* is the signal length, $w_{i}$ is the signal weight, *K* is the spectrum length.

Since a noise-free target vibration response cannot be directly obtained during real mobile measurements, it is difficult to quantitatively evaluate the denoising performance using mobile measurement data. In this study, synthetic signals were therefore constructed to train the dual-branch U-Net and quantitatively evaluate its denoising performance. Then, the trained model is used for denoising the real mobile measurement data. The specific training details are described in Section 3.2.

#### 2.3.2. Damage Detection

Structural vibration responses exhibit spatial continuity, and utilizing the variation laws of vibration signals within a given range helps define the void boundaries. Therefore, a spatially aware CNN is employed in this paper to achieve damage detection.

Step 1: Dataset construction and labeling. The denoised signals along the survey line are segmented into 0.1 s frames using the time-domain peak amplitude as the feature. A sliding window strategy is applied to group the measurement points along the survey line into triplets (three points per set) to represent the center point. Data labeling is performed based on the number of measurement points within the void region. Sets containing two or more points inside the void region are labeled as damaged data, while the remaining sets are labeled as healthy data.

Step 2: Damage detection. Similar to the denoising network, the damage detection network uses a dual-branch input for time and frequency domains. The time-domain branch consists of two convolutional layers and receives 3-channel input signals representing the time-domain vibrations of the three measurement points ($X_{t}\in R^{3\times L_{t}}$). The frequency-domain branch consists of three convolutional layers and receives 3-channel spectral magnitude information ($X_{f}\in R^{3\times L_{f}}$). The network outputs the predicted category for each set of data. The loss function consists of focal loss and flooding:

(16) $$\begin{array}{cc} L_{focal} & =-\alpha_{t}{\left(1-p_{t}\right)}^{\gamma }\log\left(p_{t}\right) \end{array}$$

(17) $$\begin{array}{cc} L & =|L_{focal}-b|+b \end{array}$$

where α is class-balancing weight, γ is modulating factor, and *b* is flooding threshold.

## 3. Results and Discussions

This section discusses and analyzes the problems encountered in the process of detecting internal damages using the impact response measured by the mobile Doppler platform.

### 3.1. Mobile Measurement Signal Analysis

**Figure 3:**
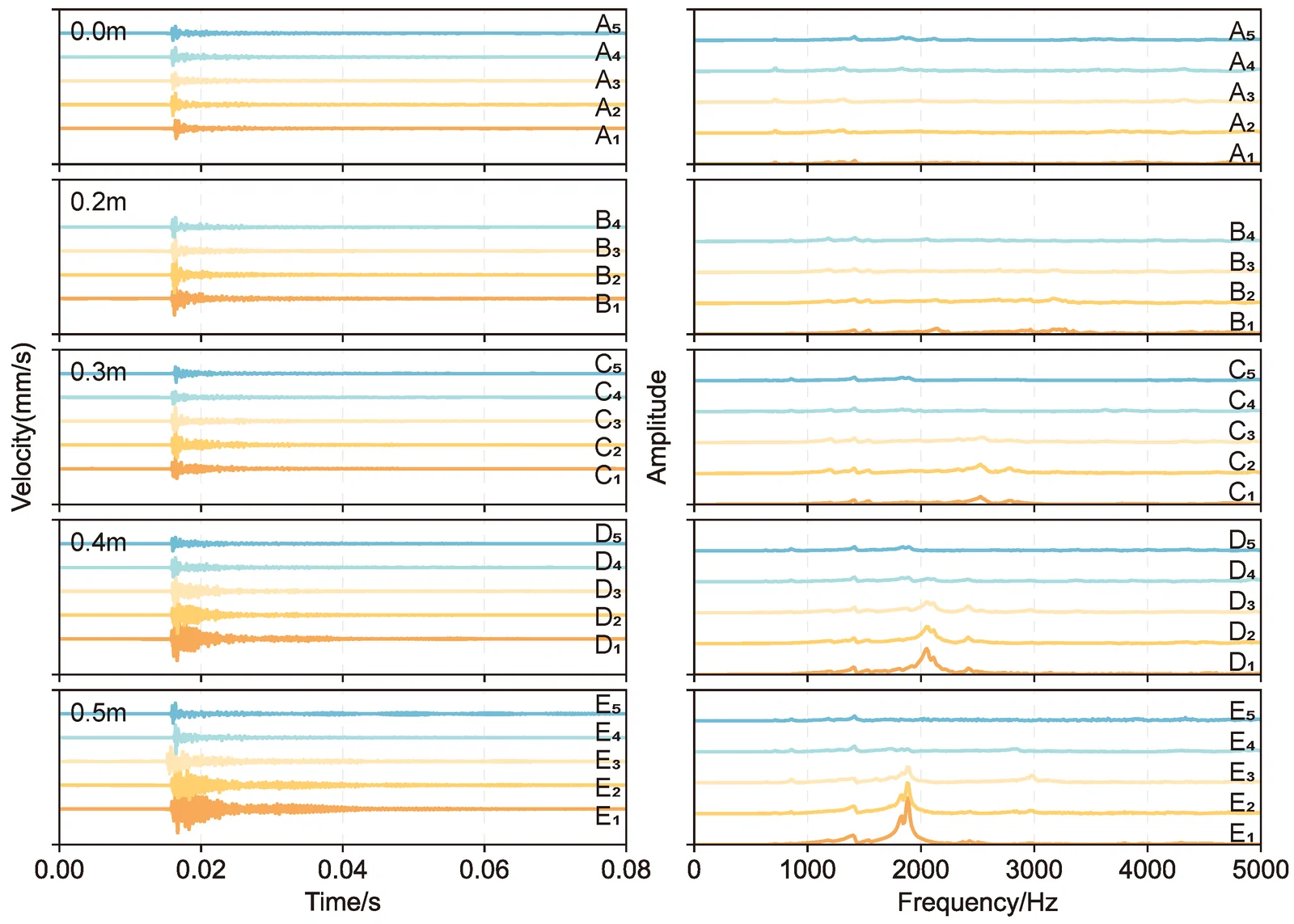
Signal and spectrum by static measurement (green dots in Figure 1).

The impact response signals and corresponding frequency spectra at different measurement points within each void region under static measurements are shown in Figure 3. The variation in the vibration signals at different void sizes is consistent with the theoretical analysis. As the void size increases, the vibration response frequency within the region exhibits a decreasing trend. For the larger voids of 0.4 and 0.5 m, high-intensity local vibration mode peaks are clearly identified in the frequency spectra. In contrast, for the small voids of 0.2 m and 0.3 m, these spectral peaks are weak, while no obvious local vibration behavior is observed in healthy regions. For the larger voids, the vibration response within the void area displays a distinct variation pattern. The vibration amplitude and spectral peak reach their maximum at the void center, where the response is dominated by the first-order local vibration mode. As the measurement point transitions from the void center toward the healthy regions, the vibration amplitude gradually attenuates, and the energy proportion of the first-order mode decreases. Additionally, excitation of the second-order mode is observed in the transition regions of 0.5 m void. But the vibration response of different voids is still dominated by the first-order mode.

As shown in the frequency spectra, the local vibration mode frequencies (*f*) under different void sizes are all above 1000 Hz. The natural frequency in Equation (11) represents the angular frequency, which relates to the frequency (*f*) in the spectrum by ω=2πf. Taking the 0.5 m void as an example, setting the first-order mode frequency to $f_{1}=1900\;\mathrm{Hz}$ (n=1) and the void length to L=0.5m, substituting these values into Equation (11) yields the angular frequency modulation relationship under mobile conditions:

(18) $$\begin{array}{c} \omega_{1}=2\pi \left(1900\pm v\right) \end{array}$$

Existing studies show that the duration of impact response signals in concrete structures typically ranges from 0.04 to 0.08 s [9,30]. To ensure the complete vibration process is captured, a sampling duration of T=0.1s is selected. The corresponding frequency resolution of the Fourier transform spectrum is Δf=1/T=10Hz. This result indicates that for the frequency shift caused by mobile measurement to reach a single spectral resolution step, the moving speed (*v*) of the platform must exceed 10 m/s. However, practical mobile impact testing is usually performed through point-by-point impact measurements. To maintain a sufficient measurement point density for detecting small-scale voids, the spacing between adjacent measurement points is generally controlled within 5–10 cm. Under a single impact and single acquisition inspection scheme, the actual moving speed needs to be controlled at around 1 m/s. This speed is equivalent to a normal walking pace and is far below the critical threshold (10 m/s) required to induce a significant frequency shift. In addition, as shown in the frequency spectra of different voids, the structural vibration frequency varies substantially across different void conditions. Even if a frequency shift of one or two spectral resolution intervals occurs, it will not lead to misjudging the void condition. Therefore, the effect of moving speed on the frequency response characteristics of measured signals is negligible. During mobile measurement, frequency changes caused by structural damage remain the primary factor influencing spectral characteristics.

**Figure 4:**
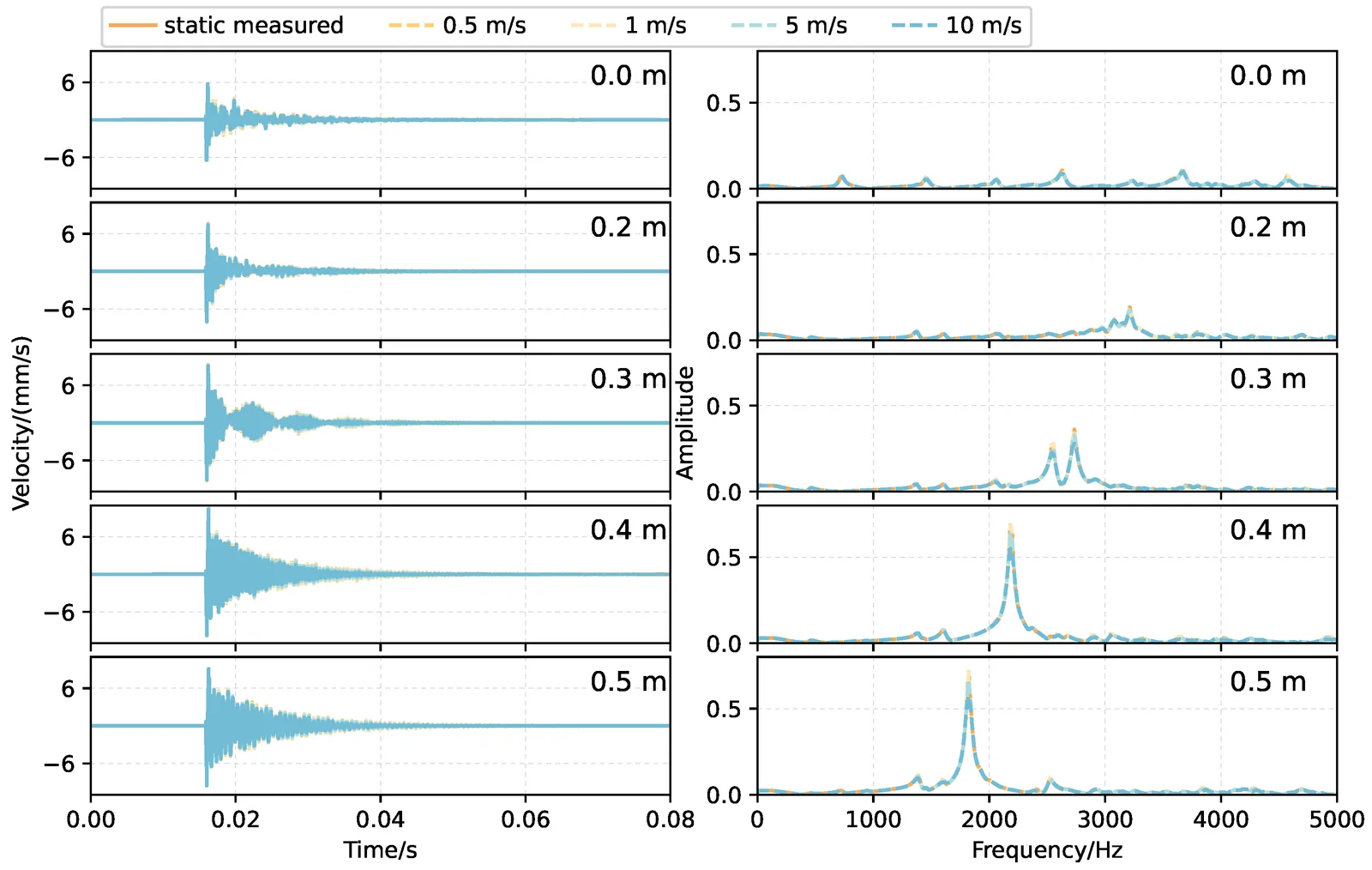
Measurement signals at different moving speeds.

Due to the presence of noise in the actual mobile measurement signal, this article uses the finite element method (FEM) to analyze the impact of moving speed on the signal, the specific modeling methods are described in detail in section 3.2 of [18]. And the mobile measurement data are obtained through interpolation using the shape functions of grid points. Figure 4 displays the vibration signals and corresponding frequency spectra at different moving speeds obtained from FEM simulations. As the moving speed increases, both the vibration amplitude and spectral peak of the measured signals decrease slightly. However, within limited moving speeds, the time-domain and frequency-domain characteristics of the measured signals remain basically consistent. To quantitatively evaluate the consistency between the measured signals at different mobile speeds and those obtained under static conditions, we futher calculated the correlation coefficient and root mean square error (RMSE) of the signal and spectrum (Table 1). The results show that, within the range of mobile speeds investigated in this study, the mobile measurements maintain a high correlation with the static measurements, while the RMSE values remain relatively low. Therefore, the influence of moving speed on the measured signal is negligible.

### 3.2. Denoised Signal Analysis

The theoretical analysis and quantitative comparison between static and mobile measurements presented above indicate that, under the low-speed conditions investigated in this study, mobile measurement does not significantly alter the main time- and frequency-domain characteristics of the structural impact response. Therefore, the static measurement data can be used as target signals to train the dual-branch U-Net. The background noise was independently collected by a moving platform over the stationary surface without applying an impact. In this case, the recorded background signals only contained platform vibration and speckle noise, without any target signals. Then, the two are combined to form the input signal for the network, and the static measurement data serves as the true value. During network training, the dataset was divided according to survey lines for cross-validation. In each fold, 4 out of the 15 survey lines were selected as the test set, while the remaining survey lines were used for the training set. The learning rate was set to 0.001, the batch size was 256, and the network was trained for 100 epochs.

**Figure 5:**
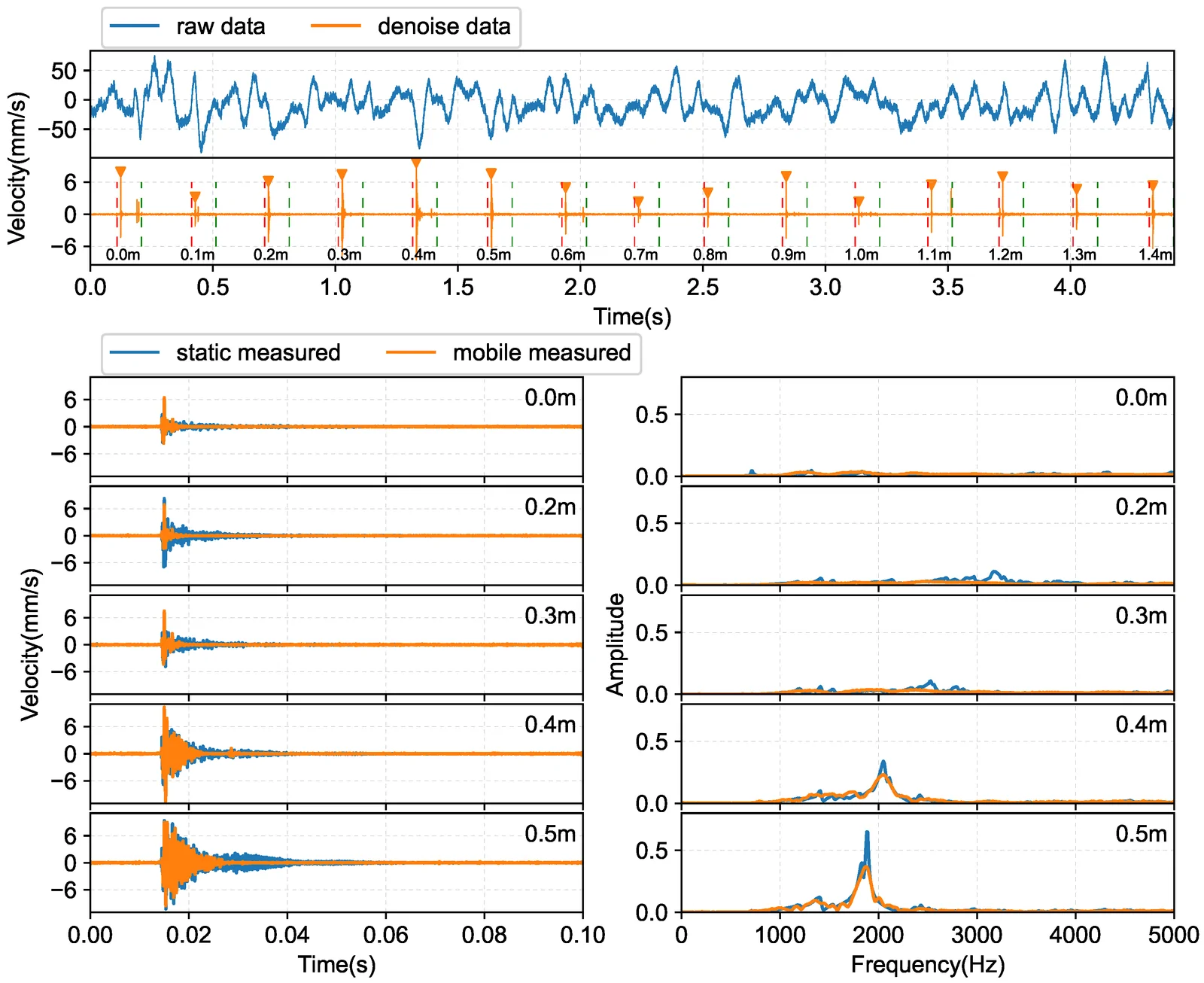
Original signal and denoised signal of mobile measurement (the red and green dashed lines represent the start and end times of the clipping signal).

Table 2 presents the specific denoising metrics in synthetic signals. Figure 5 shows the real mobile measurement signals along the survey line y = 0.3 m, the reconstructed signals denoised by the data processing framework, and the vibration signals and corresponding frequency spectra between mobile measurement signals and static baseline signals at the void location. The vibration signals are aligned by amplitude peak position. Based on our measurement data, the duration of a complete measurement line is approximately 4.5 s, and the length of the measurement line is 1.4 m. Therefore, the platform’s actual moving speed is approximately 0.31 m/s.

Due to the effects of platform vibration and speckle noise, impact vibration signals on the structural surface are heavily masked by noise. The impact response of the structural surface is therefore almost impossible to identify from the raw LDV output. After denoising via the data processing framework, both vibration signal and frequency characteristics are recovered to a certain extent. However, the recovery performance varies across different void sizes. For the larger voids of 0.4 and 0.5 m, the denoised signal exhibits a higher signal-to-noise ratio (SNR). The vibration signals show high agreement with the static baselines. The vibration decay process is effectively reconstructed, and local vibration mode peaks in the frequency spectra align well with static measurements. But the reconstruction quality of the latter half of the signal is poor, which leads to an increase in mean absolute error (MAE) and mean squared error (MSE). This is because the amplitude of the latter half of the signal is smaller and is completely submerged by speckle noise, making it difficult for the network to recover the target signal. In contrast, for smaller voids and healthy regions, the denoised signals only capture the high-amplitude response during the initial impact phase. The subsequent decay waveforms exhibit a degree of distortion, while local vibration mode peaks remain difficult to observe in the corresponding frequency spectra.

### 3.3. Detection Results Based on Mobile Measurements

The vibration signals obtained after denoising in the mobile measurements show some differences from those obtained under static measurements. To evaluate the performance of the damage detection network under mobile measurement conditions, three datasets were constructed and tested: a static-only dataset, a mobile-only dataset, and a combined static-and-mobile dataset. For the static-only and mobile-only datasets, the specimen was divided into four quadrants using the specimen center as the origin. Each quadrant contained varying degrees of voids. A four-fold cross-validation was then conducted by taking data from three quadrants as the training set and data from the remaining quadrant as the test set in rotation. For the combined static-and-mobile dataset, the static measurement data were used as the training set, while the denoised mobile measurement data were used as the test set. The learning rate was set to 0.002, the batch size was 64, and the network was trained for 100 epochs, and the final results were obtained by averaging over five independent runs.

**Figure 6:**
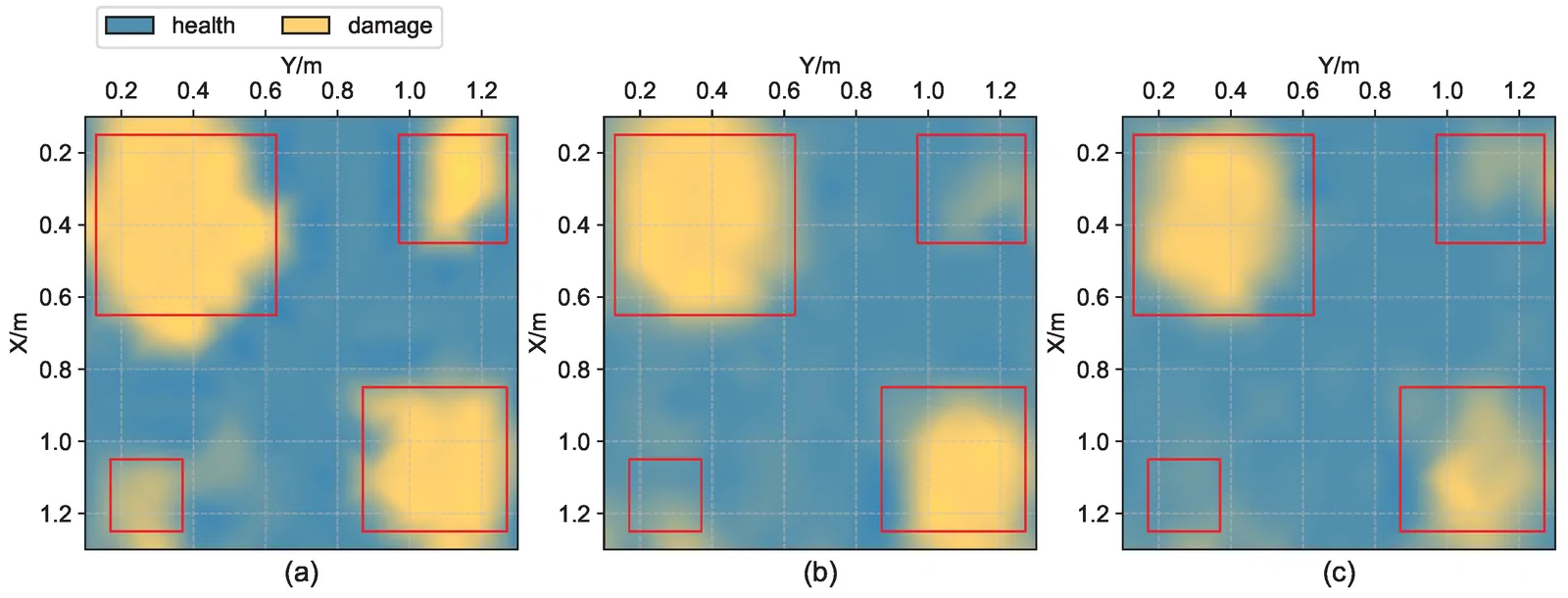
Detection results: (**a**) static-only; (**b**) mobile-only; (**c**) combined (the red squares indicate the actual void position).

Table 3 shows the quantitative metrics of the damage detection network on each fold dataset. Figure 6 shows the results of the damage detection network on different datasets. The void size has a significant influence on the damage detection performance. For the static-only dataset, the network exhibits poor identification ability for a 0.2 m void, easily leading to false predictions. Although changes could also be observed at the 0.2 m void location in the detection map, the overall reliability remained low. In contrast, the network showed relatively stable detection performance for the other three void sizes. In addition, due to the spatial characteristics of the structural vibration response, false predictions near the boundaries of the voids are inevitable, which consequently reduces the overall detection metrics. However, these boundary-related effects mainly affect the estimation of the actual void geometry and do not affect the detection of its presence. Therefore, such boundary-related misclassifications are considered acceptable within the scope of the proposed damage detection task.

For the mobile measurement dataset, the network can effectively identify only the larger voids of 0.4 and 0.5 m. The smaller voids of 0.2 and 0.3 m cannot be detected. The detection results on the mobile measurement dataset are also highly consistent with the signal recovery performance discussed in Section 3.2. For the larger voids of 0.4 and 0.5 m, the local vibration responses are relatively strong. After data processing, the main vibration waveforms and modal frequencies can still be recovered with relatively high fidelity. Therefore, the network can extract clear damage-related features from these signals. In contrast, the local vibration responses induced by the 0.2 and 0.3 m voids are relatively weak. The processed signals are more susceptible to distortion, which further reduces the feature differences between small voids and healthy regions. This limits the ability of the network to identify small voids in the mobile measurement data. Therefore, under mobile measurement conditions, the network can effectively identify only relatively large voids.

For large concrete structures, a 0.5 m void remains an early-stage defect that does not yet severely threaten structural safety. For instance, in ballastless tracks, voids generally need to develop to more than 1 m before they significantly affect vehicle ride quality [31]. In tunnel linings, voids often extend to 5–8 m [32]. Therefore, although the proposed mobile detection method cannot identify small-scale voids, it can still provide useful support for the early detection of internal voids in concrete structures.

## 4. Conclusions

To address the challenges of low efficiency and difficulty in detecting internal voids within reinforced concrete composite structures, this paper proposes a non-contact inspection framework using a mobile Doppler vibration measurement platform. The applicability of the proposed framework was systematically validated through signal characteristic analysis, mobile signal denoising, and damage detection experiments. The main conclusions are as follows:

(1) By establishing a theoretical model for the frequency shift of vibration signals under mobile measurement, the relationship between moving speed-induced frequency shift and the signal spectrum resolution was derived. Theoretical analysis and simulation results consistently demonstrate that within the moving speed range of this study, the frequency shift induced by moving speed is far smaller than the spectral resolution. Consequently, the resulting frequency shift is negligible at the investigated speeds and spectral resolution. This conclusion provides a fundamental physical basis for mobile measurements.
(2) The recovery performance of the signals reconstructed by the proposed data processing framework is related to the void size. For the larger voids of 0.4 and 0.5 m, both time-domain waveforms and main spectral peaks of reconstructed signals show high consistency with static baselines. For the smaller voids of 0.2 and 0.3 m, reconstructed signals are only partially recovered during the initial high-amplitude vibration phase, with no recognizable modal frequency peaks in the spectra.
(3) The damage detection network achieves successful identification of all voids on static measurement data. However, its performance on mobile measurement data remains constrained by the degree of effective signal recovery, and it can only identify larger voids above 0.4 m.

This study analyzed the feasibility of extending the static measurement to a mobile measurement for void detection and validated it under manual pushing experimental conditions. Theoretical analysis indicates that the current moving speed is far below the allowable upper limit. But the actual inspection efficiency depends on several coupled factors, such as platform velocity, spatial sampling interval, impact frequency, positioning accuracy, and data denoising performance. Therefore, these factors need further comprehensive evaluation to maximize inspection efficiency. At the same time, speckle noise and platform vibration are strongly correlated with moving speed. How to effectively suppress this noise is also a key research direction. Potential strategies, including optimizing the impact energy and incorporating multi-point spatial information, will also be investigated to improve the sensitivity to smaller voids. Additionally, the present validation was conducted on a laboratory specimen with a limited range of void conditions. To promote its transition from laboratory research to engineering applications, a real impact-measurement equipment and field experiments on infrastructure such as bridges or ballastless tracks are needed to further evaluate the method under practical conditions and the minimum detection threshold for the voiding range.

## Figures and Tables

**Table 1:** Quantitative evaluation results of mobile measurement.

Void		0.5 m/s		1 m/s		5 m/s		10 m/s	
		Corr	RMSE	Corr	RMSE	Corr	RMSE	Corr	RMSE
0.0 m	signal	99.91	0.042	99.66	0.082	97.76	0.210	95.53	0.301
	spectrum	99.99	0.004	99.99	0.008	99.76	0.047	98.63	0.115
0.2 m	signal	99.95	0.030	99.83	0.058	98.68	0.162	96.99	0.243
	spectrum	99.99	0.004	99.99	0.009	99.73	0.053	98.70	0.112
0.3 m	signal	99.98	0.021	99.92	0.041	99.26	0.123	97.78	0.216
	spectrum	99.99	0.003	99.99	0.007	99.85	0.054	99.06	0.136
0.4 m	signal	99.99	0.014	99.96	0.028	99.60	0.093	98.39	0.188
	spectrum	99.99	0.003	99.99	0.006	99.90	0.049	99.23	0.132
0.5 m	signal	99.99	0.014	99.96	0.027	99.64	0.087	98.60	0.173
	spectrum	99.99	0.002	99.99	0.004	99.92	0.040	99.38	0.116

**Table 2:** Denoising performance for measurement points within different void conditions.

Indicator	0.0 m *	0.2 m	0.3 m	0.4 m	0.5 m
MAE	0.12 ± 0.17	0.07 ± 0.03	0.09 ± 0.03	0.13 ± 0.08	0.16 ± 0.10
MSE	0.13 ± 0.06	0.09 ± 0.02	0.11 ± 0.02	0.14 ± 0.04	0.18 ± 0.06
SNR_before_	−16.13 ± 5.47	−16.70 ± 3.39	−16.58 ± 4.64	−13.86 ± 5.20	−12.35 ± 5.17
SNR_after_	1.58 ± 2.71	1.76 ± 2.12	1.79 ± 1.60	2.80 ± 2.58	3.42 ± 2.94

* 0.0 m means healthy region.

**Table 3:** Detection metrics.

Dataset	Fold *	Accuracy	Precision		Recall		F1-Score	
			Health	Damage	Health	Damage	Health	Damage
static-only	fold1	84.69 ± 0.02	95.52 ± 0.01	26.80 ± 0.03	87.48 ± 0.02	52.63 ± 0.09	91.31 ± 0.01	35.33 ± 0.04
	fold2	87.33 ± 0.01	90.89 ± 0.01	68.15 ± 0.05	93.93 ± 0.01	57.73 ± 0.05	92.38 ± 0.01	62.38 ± 0.04
	fold3	84.88 ± 0.02	93.90 ± 0.02	72.20 ± 0.06	83.23 ± 0.05	88.33 ± 0.05	88.10 ± 0.02	79.12 ± 0.02
	fold4	79.88 ± 0.01	82.09 ± 0.02	78.45 ± 0.04	75.45 ± 0.05	84.10 ± 0.04	78.48 ± 0.02	81.06 ± 0.01
mobile-only	fold1	83.48 ± 0.02	92.37 ± 0.00	12.22 ± 0.03	89.40 ± 0.02	17.19 ± 0.06	90.84 ± 0.01	14.16 ± 0.04
	fold2	82.74 ± 0.01	85.46 ± 0.00	35.98 ± 0.04	95.84 ± 0.01	12.30 ± 0.02	90.35 ± 0.00	18.14 ± 0.02
	fold3	76.81 ± 0.01	82.73 ± 0.01	62.05 ± 0.02	84.51 ± 0.01	58.89 ± 0.05	83.60 ± 0.01	60.36 ± 0.03
	fold4	79.59 ± 0.01	81.93 ± 0.01	77.94 ± 0.03	74.10 ± 0.04	84.71 ± 0.02	77.74 ± 0.02	81.13 ± 0.01
combined	-	80.87 ± 0.01	83.36 ± 0.01	79.03 ± 0.03	92.20 ± 0.01	48.84 ± 0.03	87.69 ± 0.01	57.11 ± 0.02

* fold1—0.2 m; fold2—0.3 m; fold3—0.4 m; fold4—0.5 m.

## Data Availability

Data will be made available on request.
